# After the death of a friend: Young Men’s grief and masculine identities

**DOI:** 10.1016/j.socscimed.2013.02.022

**Published:** 2013-02-19

**Authors:** Genevieve Creighton, John L. Oliffe, Shauna Butterwick, Elizabeth Saewyc

**Affiliations:** a BC Injury Research and Prevention Unit, University of British Columbia, L408-4480 Oak St., Vancouver, BC, Canada; b School of Nursing, University of British Columbia, 302-6190 Agronomy Rd, Vancouver, BC, Canada V6T 1Z3; c Department of Educational Studies, Faculty of Education, 2125 Main Mall, Vancouver, BC, Canada V6T 1Z4; d University of British Columbia, School of Nursing & Division of Adolescent Health and Medicine, Department of Pediatrics, T201-2211 Wesbrook Mall, Vancouver, BC, Canada V6T 2B5

**Keywords:** Canada, Young men, Accidental death, Grief, Photovoice, Masculinities

## Abstract

Young men can have an uncomfortable relationship with grief. Socially constructed masculine ideals dictate that men be stoic in the aftermath of loss, most often expressing their sadness and despair as anger. Perhaps because of alignment to such masculine ideals little research has been done to explore young men’s grief – and chronicle the ways they think about loss, their responses and how they go about describing their identities after a tragic event. Using qualitative individual interviews and photo elicitation methods, we investigated the ways in which 25 men aged 19–25 grieved the accidental death of a male friend. The study was conducted from April 2010–December 2011. Causes of death were diverse, and included motor vehicle accidents, adventure sports, drug overdose and fights. The findings revealed men’s predominant grief responses as emptiness, anger, stoicism and sentimentality. Participants’ description of their grief responses illustrated the ways in which they struggled to reconcile feelings of vulnerability and manly ideals of strength and stoicism. We gained insight into men’s grief practices by looking at the ways in which they aligned themselves with a *post-loss masculine identity*. These identities, which included the *adventurer*, *father-figure* and the *lamplighter*, revealed gender-specific processes through which men understood and actively dealt with their tragic loss. The results offer novel insights to men’s grief and identity work that may serve to affirm other men’s experiences as well as guide counselling services targeted to young men.

## Introduction

Grief can be a challenging experience that catalyses a diverse array of social processes and practices (Jacobs & Prigerson, 2000; Ritchie, 2003). While there has been scholarly attention paid to grief and the linkages to health and illness, gender analyses are conspicuously absent and, in particular, studies examining connections between masculinities and grief among young men. Instead much of the literature has focussed on describing gender differences between men and women. When Western men grieve in ways that invoke stoicism, anger and rationality, it has often been explained as flowing from socially sanctioned masculine ideals (Martin & Doka, 2000). Inversely, emotional outpourings, such as crying, expressed by Western women in grief are conceived of as typically feminine behaviours (Martin & Doka, 2000; Shamir & Travis, 2002). In the specific context of bereavement induced grief, Archer’s (1999) review of the literature revealed that men experience significant mental and physical health impacts following the loss of a spouse, with subsequent mortality was most often attributed to accidents, lung cancer and heart disease (Martikainen & Valkonen, 1996). W. Stroebe and M. S. Stroebe (1993) suggest that this may be due the tendency for men to have fewer social support networks than women do. In contrast, Archer (1999) found that many men recover from grief more quickly than do women. Nolen-Hoeksema (1997) suggested that men’s “problem solving” approaches to grief can reduce their potential for developing reactive depression.

Because expressions of grief are deeply gendered, they are also powerfully policed and men who grieve in ways that do not embody socially assigned masculine practices (such as stoicism and rationality) can feel judged and alienated (Martin & Doka, 2000). The social practices around men’s grief have been deemed detrimental by Zinner (2000) because “manning-up” and adopting a form of toughness positions crying and/or seeking supports as being weak and un-masculine. Perhaps this is especially evident among young men who aspire to embody manly virtues of competitiveness and self-reliance and risk taking following the loss of a significant other (Archer, 1999; Davies, McCrae, & Frank, 2000; Mayne, Acree, Chesney, & Folkman, 1998). Rieker and Bird (2000) have referred to such practices as choice disability, arguing that gender restraints can constrain men’s expressions and perhaps experiences of death related grief.

The aim of this article is to describe young men’s grief experiences and how they expressed a masculine identity following the accidental death of a male friend. Findings from the study, while potentially affirming other men’s experiences of grieving, may also influence young men-centred counselling services.

## Masculinities, young men and death

The leading cause of death for young Western men between 19 and 24 years is accidental injury (Kelland, 2011; Phillips, 2005; Statistics Canada, 2005). Many young men are killed in motor vehicle accidents, and these fatalities are often connected to recklessness, excessive speed and impaired driving (ICBC, 2007). Other leading causes of mortality include sports related and workplace deaths along with unintentional substance overdose (Statistics Canada, 2005). These longstanding tragic outcomes have, to some extent, been positioned as a fait accompli in the lives and deaths of many young men. Sex based explanations have posited evolution (Maccoby & Jacklin, 1974; Wilson, 1975; 1978), hormones and brain physiology (Kemper, 1990; Maccoby & Jacklin, 1974; Steinberg, 2007; Wilson, 1975) as biological drivers for men’s risk taking, violence and involvement in extreme sports (Campbell, 1999; Pawlowski & Atwal, 2008). Similarly, Cobb (2004) argues that male adolescents are not developmentally mature enough to understand the consequences of risky actions.

More recently, attention has been paid to how social constructions of gender (and their intersections with other social determinants of health including culture, social class and socioeconomic status) influence an array of men’s health practices including risk-taking. Key to this men’s health work has been the adaption of Connell’s (1995) masculinities framework, which is based on two principles: (a) patriarchal power and characteristics, including stoicism and self-reliance, which are understood as hegemonic masculine ideals that influence men’s affect and health practices, and (b) a plurality of context-dependant masculine performances are embodied by men in relation to hegemonic masculinity.

Connell (1995) masculine performances are categorized as complicit, subordinate and marginalized; by definition, complicit masculinity sustains hegemony by enacting social practices that approximate or reproduce men’s hegemonic status in the social hierarchy. In the context of the current study, the masculinities literature suggests that many young men are complicit in sustaining hegemonic masculinity by engaging in high-risk activities (Connell, 1995; Grieg, 2009; Messerschmidt, 1993; Robertson, 2006), practices which result in many seemingly preventable accidents, injuries and deaths within that sub-population of men (De Visser & Smith, 2006; Frosh, Phoenix, & Pattman, 2002; Kimmel, 2008). Subordinate forms of masculinity are associated with failed hegemony, for instance, lack of authority, weakness, domesticity, and statuses associated with femininities such as emotionality and dependency. Marginalized masculinities are linked to de-privileged race, class and ethnic markers, and include men who are excluded because of their perceived deviation from standards of white Western idealized masculinity (Connell, 1995). In the context of the current study, subordinate masculinities may be assigned to young men who express their grief though crying and/or who become careful and conservative rather than risk-reliant because they fear future injury. Young men are also exposed to a variety of masculine ideals and, for marginalized men, the options may be narrower based on personal characteristics (e.g., tendency towards risk-taking), cultural milieu, and personal circumstances (e.g., growing up in poverty, absent fathers, high-risk environment, etc.). Young men have variable agency and levels of choice disability in rejecting, reconfiguring and aligning to specific hegemonic ideals as a means to demonstrate their complicity or contesting their subordinate or marginalized masculine status.

## Methods

The methodological approach informing our study was interpretive description, a qualitative method drawing on the explicit logic of how knowledge is used in the applied health disciplines (Thorne, 2011). Our approach relied on a knowledge-to-practice orientation whereby we sought out knowledge with the intention of garnering empirical findings that could be utilized to improve the practice of health care providers and support workers who come into contact with men who have experienced loss (Thorne, 2008). In keeping with this approach, our social constructionist framing was purposely employed to produce knowledge with the aim of understanding young men’s grief as a means to positively influencing targeted support and counselling services. Data collection methods, including individual interviews and photo-elicitation, were used to explore how participants grieved the accidental death of a male friend amid describing their post-loss masculine identities. Integral to our approach was attention to the meaning that participants made of their stories and photographs, drawing connections with material, social and institutional conditions. Addressing the overarching research question, *How do young men grieve and construct masculine identities following the accidental death of a male friend?* we inductively derived and developed findings based on the young men’s interviews and photographs.

### Sample

Twenty-five men, ranging in age from 19 to 25 years old (mean = 21), who had experienced the death of a male friend within the last three years participated in the study. Participants were eligible for inclusion if their friend’s death was due to a risky activity (considered risky by the researcher—not necessarily the participant) such as drug overdose, motor vehicle accident, sports injury or fighting. Participants were Anglo-Canadian (*n* = 14), and Aboriginal (*n* = 5), South Asian (*n* = 3), Central American (*n* = 1), Jamaican (*n* = 1) and Bermudan (*n* = 1) and resided in Vancouver, British Columbia, Canada and its surrounding suburbs. Additional sample demographic details are included in Table 1.

### Data collection

Following University ethics approval, participants were recruited using posters, flyers, online advertising (Facebook and Craigslist) and word of mouth. Information regarding the project and its aims were distributed to staff of local youth serving organisations to highlight our research and direct study information to potential participants. Potential participants could request more information or volunteer for the study by contacting researchers by telephone, text or e-mail. The data collection methods included two semi-structured interviews using photo-elicitation techniques. Upon completion of the written consent and the collection of demographic data, the first interview included open ended questions that invited participants to share details about the accidental death of their male friend[s], as the foundation to describing their relationship to the deceased and initial responses to the death. Towards the end of the first interview participants were invited to complete a “photo assignment”, producing a series of photographs to illustrate their grief and post-loss activities. Some prompt questions were provided to guide the men’s picture taking (please see Table 2). In the second interview participant-produced photographs informed additional questions and prompts to further develop understandings about the connections between masculinities and young men’s grief and post-loss identities. We also invited participants to share their perceptions about how masculinity and being a man informed their pictures and influenced their reactions to the accidental death of a male peer.

Participants were given two weeks to complete the photo assignment, at which point a second interview was scheduled. The participant brought the camera to the meeting and pictures were loaded onto a laptop computer so that they could be viewed by both the interviewer and participant. The second interview was driven by the photographs that participants had taken.

Data was collected in 2010 and 2011. Interviews were conducted by a trained female researcher (first author) and participants received $20 honorariums following each interview, to acknowledge their contribution to the study. Participants were provided with contact information for professional grief and crisis intervention services. Interviews were digitally recorded and transcribed-each participant was anonymised by removing all identifying information and the men were given a pseudonym to link interview excerpts to individuals.

### Data analysis

Photographs were initially loaded into the digital folder designated for each participant (which also contained digital recordings of the interviews, interview transcripts and field notes). Photos were then imported into Atlas.ti6^™^ each as a primary document. General notes about the context and basic content of the interviews were attached to each primary document as free memos. Photos were electronically linked (within Atlas.ti6^™^) to participant interview transcripts. Descriptors of photographs present in the interview transcripts were noted and attached as memos to the corresponding image.

Interviews and their corresponding photographs were analysed using constant comparative techniques whereby textual and image based “units of meaning” were reviewed to develop and refine themes as well as to explore relationships and patterns across them as a means to integrate and make sense of the data (Bong, 2002; Corbin & Strauss, 1990). We also did a narrative analysis, which involved examining each interview in its entirety to discern an overarching theme. Employing these approaches in tandem ensured close examination of the elements of each participant’s story and the themes that were common (or not) across interviews and images. While the photo-elicitation was included to garner conversation and facilitate men’s reflection within the interview (Oliffe & Bottorff, 2007), participants’ photographs were also used to explore narratives about grief and masculine identities. Included within the findings are some illustrative photographs and their corresponding narratives.

## Findings

The findings are arranged to in two sections to share men’s immediate reactions and more down-stream effects. First we detail the men’s accounts of their grief in response to the news that a male friend had unexpectedly died. Second, these accounts of grief are examined in the context of how they reflect particular masculine identities in the aftermath of that loss.

### Emptiness and stoicism

Many men described feeling empty or hollow in the time immediately following their friend’s death. Embedded in these narratives were expressions of shock and uncertainty about how to react; in this respect, men’s emptiness emerged both as a byproduct of their male friend’s death and an inability to be action orientated in their immediate response. Participants typically described an intermediary period between hearing of the death and an emotional response in which they experienced unfamiliar disabling immobility and passivity. Damien and a few close friends were on their way from a pre-party to a school sponsored grade 12 graduation celebration. Neither wanting to pay for a taxi or drive intoxicated, the friends opted to hitch a ride in the back of a van. When the van stopped, Damien’s friend jumped out to run across the street to the event. In his haste, he did not see the bus intersecting his path. The teenager was struck and killed in front of Damien and his twin sister as well as the other young party goers across the street. Damien recalled being taken home in a taxi at midnight following hours of courthouse interviews, his friend’s sister screaming hysterically beside him. He took Photograph 1, which showed an empty bucket to illustrate how he felt in the days and months following the accident.

The only thing that I could really think of was an empty bucket because that is how I felt; just so empty and hollow inside and I just didn’t really know what was going on.

Most men understood this emptiness as making them vulnerable to uncontrolled emotions, some of which might potentially emerge as un-masculine expressions of grief such as crying and/or irrational muddled thoughts and speech. A poignant example of this was shared by Joe, a 22-year-old man whose friend had died when he fell through a skylight while climbing on the roof of a house during a party. He recalled a steadfast desire to be strong in the midst of this tragedy but he was unable to embody such masculine ideals.

I’ve always been able to be the calm one, the one people could depend on to be rational in any situation and to bolster other people.

He shared Photograph 2, an image that depicted his vulnerability and in describing the house as a frame with half built walls and an open roof he drew comparisons to how he felt following the death of his male friend.

I took this because I was like… after my friend died, it’s like, well, that protection kind of comes off and you’re more exposed.

The vulnerability that Joe and many other participants referred to suggests that manly virtues of strength, decisiveness and self-regulation were disabled by their sudden losses, in ways that left many men unable to publically align with such masculine ideals. These stories, among several, highlight the dominant socially constructed ideals about how Western men can do grief. Participants’ concerns with being seen as less of a man led many young men to hide themselves away until they regained their composure. To counter vulnerabilities men tended to remain solitary and stoic in order to reinstate some control as to what could be seen and potentially judged by others. In addition, time alone afforded refuge for sorting through un-masculine feelings of sadness and despair privately.

Men’s stoicism was rationalized by some participants as *inbuilt* whereby, regardless of the sadness that might be experienced, the male body self-regulated outward expressions of grief. Shawn, a 19-year-old, lost his friend to a motorcycle accident. Following a pre-graduation party his friend boarded his brand new motorcycle impaired and drove towards home. Hitting a patch of gravel next to the highway, he lost control of his bike and struck a telephone pole. Reflecting on the aftermath of the accident, Shawn went onto explain that he, like most men, is unable to cry.

“I can just feel something in my stomach that feels terrible and it just won’t connect with the tear glands and I think that’s just how guys are.”

Nathan, a 22-year-old man recalled hearing the news about his friend’s death. While detailing how his friend was stabbed in a fight outside of a bar that fateful night, Nathan provided assurances that men’s control over a tearful response goes beyond biological impulses. Men learn to control their expressions; most revered as a manly virtue is the strength to maintain that control and align to those masculine ideals regardless of the circumstances:

“That’s how you’re taught, that’s how you are brought up. Men are taught from an early age: don’t cry, it’s not your job… How are you going to be strong if you are crying it out?”

In response to this perceived norm, most participants agreed that “manning-up” was best embodied by taking actions towards controlling their affect. For example, Nathan argued that men need to “fight through it” and Dylan, a 21-year-old explained the need to “turn it down” while Damien in referring to Photograph 3 asserted that he felt compelled to “Turn it off”:

Implicit also to the men’s narratives and photographs was the notion that stoicism and emotional restraint could afford some self-protection. So while masculine norms informed many men’s responses and actions there was also concern that ‘feelings’, felt and/or expressed, could lead to dangerous levels of introspection, a pathway that decidedly strayed away from strength-based masculine ideals to which they subscribed Photograph 4 and 5.

### Anger

A few participants described being enraged by their friend’s death, but such affective reactions were contextually dependant. For example, Aiden and his group of male friends reacted strongly to a friend being shot dead by police intervening in a domestic dispute. Gripping the table between us in the interview, Aiden’s fiery response was palpable in his assertion that anger was a legitimate masculine way to deal with the preventable death of his friend:

It’s a stupid male thing, but because it was a violent death, I felt a lot of retribution and revenge. I was consumed with anger and [the girls] went straight to the sadness, not to all the anger and stuff.

Aiden acknowledged his fleeting interest in avenging the death of his friend as a form of acting out, later on suggesting that some men actually do take that course of action in the heat of the moment. As Kilmartin (2007) reminds us, anger is one of the few losses of control that men are routinely afforded as a manly expression. In this respect Aiden’s angry talk, though not mobilised towards violent action, was an acceptable manly way to contest authority and injustice in the context of losing his friend. In addition, Aiden’s vengeful response was clearly differentiated from the sadness which consumed the women around him.

Others experienced anger as a counterpoint to sadness. Ben, a 20-year-old who lost his friend in an accident, explained his anger over the circumstances of his friend’s death. In the midst of his despair over the loss of a young life he was enraged by his friend’s decision to consume alcohol before getting on his motorcycle to drive home:

It angered me, not for the fact that he passed away, but for the fact of how it happened. It’s one thing to pass away on a motorcycle, but it’s another thing if there is a different conclusion, different factors and variables playing into it, right? It’s selfish.

For Ben, anger over his friend’s death reflected and focussed on the futility and anguish of a preventable death whereas Aiden’s reactive anger was less controlled and considered in being directed towards the perpetrators (i.e., the police). Within both examples, however, anger was understood as an emotion men may legitimately experience and express.

### Sadness

An intense emotional response of sadness was described by some men, and for the most part this was positioned as a potential site of vulnerability. Many men’s displays of emotionality emerged from unfinished business whereby they lamented what they wished they had said to their friend or done to prevent his death. These participants often dwelt in regret, wondering what they might have done differently. Alex, now 25 was 23 when his friend died after driving his truck over an embankment. He heard news of the death while he was at work and remembers going to his car and spending the night in the parking lot, unable to drive away:

We hadn’t spoken for a while because we had a disagreement. I guess we were estranged. I just felt so horrible inside, like I could barely breathe, and I haven’t been able to shake that. I always thought we’d repair our friendship and it would be like before. Now we never will. It’s just over and there’s so much I can’t say.

It is important to recognise that the participants who acknowledged their sad, emotional response were most often born outside of Canada and/or had parents who had immigrated to Canada. The connections between masculinities, culture and grief suggest that, among this subgroup, it may have been more acceptable to express their sadness directly. Moreover, these participants did not position being a ‘man’ or being ‘masculine’ as necessarily rejecting or controlling feelings of sadness. This was illustrated by Amir, a 20-year-old, who came to Canada as a refugee from Afghanistan when he was a young teen. While steeped in the dominant cultures of his Western peer group, his household maintained the traditions and practices of his Eastern birthplace. As Amir spoke about his sadness, his eyes filled with tears and his voice quivered as he explained that he had actually lost three close friends. While assuring us he had moved on, enjoyed socializing and had lofty career goals, his sadness was ever present in recounting the intensity and long term impact of what he felt:

I feel, oh God, why do you always take the good ones? I feel sad. Like sadness is coursing through my veins.

### Grief and masculine identities

Participants downstream grief responses emerged in relationship to three predominant masculine identities and we inductively derived three archetypes; the *Adventurer, Father Figure* and *Lamplighter*. The three predominant identities were labelled using phrases from representative participant interviews, and are presented in order from the most to least common. That said, it is important to note that while individual participants tended to embody a specific identity they are not espoused as fixed nor entirely devoid of other identities.

### The adventurer

The ‘Adventurer’ identity was embodied by men whose performances were enacted through journeying out into the unknown—experiencing new cultures and making one’s own way in the world. Included were daring activities such as mountain climbing in Nepal, surfing in Thailand and skiing in Europe. Grief, for these men, catalysed action. For example, Chris, a 23-year-old, had a friend die in a mountain bike accident. He understood and described himself as a man of action who was willing to embrace dangers to ensure he experienced all that life had to offer:

I’m a relatively outdoorsy person…I was just in Iceland, and we actually walked up the glacier while it was completely, completely windy and snow was falling out on us, and I think we didn’t know if this was okay, if people had done it before. But we just wanted to get a really great shot of the volcano. I was there with a guy who was studying as a volcanologist, so we kind of just took the risk of, like, you know, that the ice won’t crack and that we’ll be actually able to just walk through it, and we won’t fall off the large glacier itself.

Chris was off travelling when he heard of his friend’s death. While he was initially struck with sadness he remembers the death as feeling “very far away”. Rather than returning home to attend the funeral, he continued to travel for several more weeks. He stated that the way he continued to embrace life was the best way of honouring his friend.

Chris, like other participants, described his life as primarily driven by a desire to engage in new and exciting adventures. Once an experience had been had and/or the goal had been accomplished these men looked towards their next conquest. Their lives were transient by design. These much sought after experiences demanded that the adventurer was bohemian and mobile, able to live sparsely, and find refuge in a tent, bachelor pad, communal staff housing or on a friend’s floor. Indeed, a feature of the adventurer was his ability to adapt to the availability afforded by any range of unconventional circumstances.

Participants who adopted an adventurer identity valued freedom and they were attached only momentarily to any one landscape, person or activity. This adventurer orientation towards separateness and perhaps solitariness also influenced connections to family and friends. Adventurers connected to like minded individuals to share and witness snippets of their adventures. But, ultimately the narrative of the adventurer was characterised by hedonism and an insensitivity to others. Engaging in public expressions of mourning with other friends was situated as an uncomfortable and even suffocating experience.

Associations with women also figured strongly in the adventurer tales, and again, these connections were typically experiential and short—time focussing on physical rather than emotional relations. For example, Daniel, now 23, was 21 when his friend died of a drug overdose while on a surfing trip in Australia. He spoke extensively of the life he loved to lead, a surf culture featuring women, drugs and alcohol:

Yeah, so I met these girls on the beach and we did some acid, and then somehow we end up at a house all together in this bubble bath. The girls were both strippers and they stripped for me. I ended up taking some pictures.

In Daniel’s account, and common to many men’s adventurer tales, the death of their male friend sustained or gave rise to an array of hyper masculine performances. The adventurer performs these hegemonic ideals of autonomy and bravery alongside invulnerability to pain and grief. While purportedly unconcerned by the material necessities of the mainstream, the masculine performances of the adventurer were typically supported by well-heeled personal and/or family socioeconomic circumstances that facilitated their lifestyle.

There was some evidence to suggest that, although adventurers’ plans to experience all that they could were not entirely new, the unexpected death of a male peer fostered fatalism and garnered men’s drive to do more rather than less in this regard. Adventurers identities understood the death of a male peer as an ever-present risk in doing the things that young men need and love to do.

### The father figure

The father figure identity provided a sharp contrast to the adventurers, in that their central consideration was honouring their responsibility to care for friends and family. Embedded in these men’s narratives were references to manly virtues of loyalty and the protection of others. Some participants spoke of being a man in a similar way that one might speak of divinity with the power to created and care for that which you are responsible. Ben, age 20, whose friend was killed in a motor vehicle accident, explained his virtuous desire to be responsible for his actions:

I think being a man is just about taking responsibility and pride for what you as a person are putting into the world. That’s what being a man is, you care [for] your relationships with women, you care for your children, and you care for your ideas. You need to care for your job, you need to care–I mean, it’s all good to not care, but you need to care about the right things, and for me, simply put, I’d say it is about caring for the things you put in the world.

Similarly, after his friends’ death, also as a result of a motor vehicle accident, Shawn, a 19-year-old man strived to be a “strong-minded person that is there for more people to rely on, more than for me to rely on people”. Key performance indicators, for these men, involved sustaining a strong network of family and friends while maintaining their health and achieving academically and/or career wise as the conduit to being a family protector and provider. While acknowledging the potential of the lumberjack in photograph 5 to be ironic or parody, Damien, a 21-year-old man articulated the masculine ideals to which he aligned in responding to the question “What symbolises being a man to you?”

I can only think of one and it’s this guy, the Squamish lumberjack. I wanted to get a picture or two of my stepdad and my mom and my sisters in front of the house… just like, as a provider, a husband, a father, a family man, all those responsibilities tied into one as well as a lumberjack. Being stronger and tougher and that kind of thing.

Men describing a father figure identity embraced the sturdy oak masculine ideal in the aftermath of losing a male friend in ways that were intent on recognizing, and not adding to, the grief of others who had been directly impacted. In addition, there was a strong desire to ensure their own actions would not lead to their death and the grief related pain they had witnessed in others.

This was particularly the case in men’s actions towards ensuring the well-being of the women in their lives. Some men positioned their strength around the death of their male peer as supporting others and controlling the emotional outpourings that might flow from such tragic circumstances. As Joe, a 22-year-old man who lost his friend when he fell through a sky light explained: “You don’t want to trigger other people. When they’re trying to deal with… and if they are dealing with it in a different way you, don’t want to, you know, step on boundaries.” Unlike the Adventurers, the Father Figure privileged connection and responsibility.

The care and loyalty that these participants expressed towards others extended to the memory of their peer who had passed on. Honouring the friend’s life sometimes called for men to defend their friend’s death. Concerned about the potential for stigma or generalisations that their friend might have been just another young man who took thoughtless risks, many men were quick to assert that it was a case of being in the wrong place at the wrong time. In response to a question about whether or not he considered his friend who died to be ‘reckless,’ Joe talked about the distress he felt when others assigned such negative labels:

How do you label somebody as reckless? How do you label anyone after they passed away? Like how do you, like why should you be able to you know? It’s hard when you know the person, like how are you calling that person something; you don’t know that person, right? You don’t know about their personality, you know, so it’s odd having someone putting a label on someone that you know.

Evident in Joe’s, and many other men’s assertions, was the need to refute any efforts towards victim blaming. In this respect the men’s protection extended to ensure the life (rather than the circumstances of their death) and memory of their friend was honoured.

### The lamplighter

The Lamplighter identity entailed a rebirth of sorts whereby the hardships and struggles gave rise to an archetypal hero’s journey. A small group of men explained that they had experienced first-hand the substance overuse and delinquency that ultimately claimed their friend’s life. However, the death of their friend led them away from those practices and inspired, in part or whole, their life change. Moreover they wanted to be role models for helping other men to embrace similar changes.

Symbolically, these participants ‘saw the light’ when their friend passed away. The death gave them pause, prompted introspection about their own lives and catalysed their efforts towards changing for the better. Noah a 21-year-old and his best friend played in a heavy metal band together with four other young men. Along with making music together, the band spent much time partying, with drugs and alcohol ever present. On many occasions rehearsal times would be cut short when they decided that the evening would be better spent testing the bounds of the law. On one occasion, Noah was arrested for speeding and driving under the influence in a car he had stolen. Everything shifted when one of Noah’s band mates, also a guitar player, died of a drug overdose. He talked about how his friend’s death catalysed an immediate change in him:

When I was young I was a troublemaker, to say the least. When [friend] died, it was a transformation period for me because I was getting out of all of the bad stuff and learning how—I was learning more about myself than about everybody else. I was introduced to a lot of good people in that year, and pretty much from there my life took off for the better… When I’m older, I want to be that person for somebody else.

Ethan, a 24-year-old man and his friend also bonded over the daily use of an assortment of mind altering substances acknowledging that “I was addicted to alcohol and he was addicted to drugs”. After his friend died of a drug overdose, Ethan recalled being outside of the funeral home and reflecting on his own existence as an epiphany to avoid a similar outcome:

I just looked at my life and it’s kind of, if I were to go today, like what would I have to show for myself and what I did with my life? I felt like I was having a good time, but I wasn’t fulfilled in terms of happiness or anything like that. I just wanted to be happy and wanted to be able to touch people like (friend) that are going through a hard time and let them know that it’s not as bad as it seems.

Perhaps Levi, a 23-year-old illustrated the lamplighter identity best in photograph 6, amid chronicling his impoverished background, violent family members and the recent death of a friend who was stabbed outside a Vancouver nightclub:

This is a lamppost and it was daytime, so it wasn’t an honorary thing, but I guess it was a bit of an extension of the other pictures, because I want to kind of be a light to younger kids, but I’ve also had a number of mentors who’ve been a light to me.

The lamplighter’s circumstances tended to be modest in terms of direct family supports, and in some respects the male peer deaths that they chronicled were implicitly, and sometimes explicitly, positioned as inevitable given the socioeconomic hardships the men shared. Said another way the lamplighter stories of these deaths occurred amid contexts of violence, poverty and fractured families, from which recurrent, multiple exposures to losing a male friend were often endured. These participants and their friends were arguably marginalized masculinities, empowered as self-proclaimed “bad kids” in their own communities but without economic capital and social position to achieve broader ideals of hegemonic masculinity. Friends could be said to have died in the performance of rebellion or protest e.g. drug overdose, knife fighting and shot by the police during a domestic dispute.

Despite a life of abiding hardship, these participants were neither vengeful nor complacent. The masculine virtues they sought to embody and impart, in a departure from those that they previously idealised, were generative, active and instrumental. They had mobilised the experience of having a friend die towards ensuring that the tragedy might save others from enduring similar hardships. The lamp symbolically draws its energy from the grief over the friend who has died to catalyse change for good not evil. Levi, a 24-year-old man explained that his goal was to positively influence the perpetrators as well as the potential victims, because that strategy would most likely break the cycle:

Part of what I want to do is try and be a tech teacher and try and influence the young kids who grow up to stab people at night clubs. I don’t think they were born that way, I believe it was because of the circumstances, and mostly the people that they have around them. So whatever I can do to counteract this I kind of see as a tribute to him, because when I was first thinking about it, what had happened to him, I didn’t really have a lot of anger for the people that did it. It was more like, I don’t know who they are but they probably weren’t surrounded by the best people while growing up.

The goal of the lamplighter was to sustain their changes in honouring their deceased friend. Whether it was their own personal commitment to be sober, or loftier goals to assist other individuals and/or their community, the lamplighter subgroup were focussed on being visible in the world as a shining light and example that men can reinvent themselves.

## Discussion and conclusion

Unintentional injury is the number one risk to the health and wellbeing of young men in North America. Barth (2001) has referred to young men between 15 and 25 as the “dangerous demographic” because of the elevated mortality in this group, as a result of injury due to car accidents, reckless behaviours and violence (Statistics Canada, 2005). While a majority of deaths occurring among young men are sudden and accidental, there is a paucity of research exploring the impact of these deaths on their male peers. Our study findings address this knowledge gap by making available an array of reactions and masculine identities that emerge in and around the tragic losses that so often occur among young men. Evident were young men’s vulnerabilities that flowed from their profound unexpected losses and accompanying participant’s words were a collage of highly revealing photographs. Within the men’s grief processes were the influence of masculine ideals that guided how they might reasonably grieve in public when the events were fresh in participant’s minds. Stoicism and anger can all be explained away as masculine ideals to which the men could legitimately align. We do not intend to argue that embodying these masculine ideas was either positive or negative for the health of participants. On the contrary, outpourings of emotion do not necessarily foster a ‘better’ experience of grief, and given the young age of the participants – relying on the aforementioned masculine ideals may have afforded some familiar performativity terrain to ease the sense of profound loss.

While a minority of men talked about the tears that accompanied their sadness, most men spoke of the act of crying, particularly in a public outpouring of grief, as a feminine activity that would be seen as unacceptable or as signifying weakness to their friends. It is evident in the literature that women as well conceive of crying as feminised (Archer,1999; Martin & Doka, 2000; Versalle & McDowell, 2004–2005). This gender policing of grief, the social dictate to “man up”, has consequences for men. Restricted options for processing and expressing grief led men to engage in activities in an attempt to mask feelings or make them go away. Following the death of their friend, for example, most participants spoke about the ways that they engaged in health harming behaviours in the form of substance overuse, driving fast while under the influence or doing sports such as skiing or climbing without taking safety precautions.

In terms of masculine identities it is fair to say that, given the age and the temporal proximity of our interviews with the men’s death of a peer, there is much that might change in how participants idealise themselves as men across time and their life course. Related to this we are limited in what we might claim flows entirely from the loss of a male peer versus what emerges at varying time points in young men’s lives. For example, men who described having an adventurer identify may have been likely to take on that identity independent of the death of their friend, while there was some urgency among the lamplighters to avoid the all too common deaths within their impoverished social group. The father figure in turn aligned to honourable masculine virtues that perhaps signalled a loss of innocence and the need to be mature beyond their years.

Men’s gender work is dialectic, whereby opportunities are present for some men to reinvent themselves as changed for the better – in part at least - as a direct result of their loss and grief. While certainly not exhaustive these masculine identities are deeply connected to men’s social location and illustrative of the ways in which masculinities shift - processes Connell (1995) refers to as a gender project. The father figure steps into adulthood and, as the term father implies, also receives the privileges of complicity masculinity. It is less clear in the case of the lamplighter who, while engaging some of the virtues that signal a hegemonic masculinity of the middle class, is still socially located within a working class/marginalized context. The adventurer maintains his masculine practices, reproducing those of his peer group-elite by way of social and economic capital. This highlights the way that all these masculine identities are contextually linked to other social determinants of men’s health in ways that might restrain men’s choices or afford an array of options – depending on how the men think and engage with *their* dominant discourses of masculinity. In this way, our study goes some way towards addressing Hearn’s (2010) recommendation to address the gendering and embodiment of youth and young men to fully understand the contradictory means by which men receive status and experience marginalization. However, future research might benefit from adapting intersectionality to more explicitly engage other social factors, such as social class and ethnicity (Griffith, 2012).

Practically, findings from this study can inform the formal practice of health care providers and youth workers and the more informal supporters in young men’s lives such as parents, coaches, friends and teachers. An understanding of the barriers that men might to outward expressions of sadness and loss can inspire more attention to other ways that they are communicating that they are in distress. Clinical services can be adapted to affirm a wider array of grief practices including ones influenced by dominant ideals of masculinity.

This study also affords methodological insights to guide the efforts of future men’s health research. Oliffe and Bottorff (2007) suggested photo-elicitation can uniquely engage men in qualitative research and topics with which they are not expected to articulate to others. Our experience of conducting this study resonates entirely, and we were struck by the depth of the men’s thoughts amid their creativity round the images and narrations they shared. While photo-elicitation has been used to detail men’s experiences of prostate cancer (Oliffe, 2003) and smoking through the eyes of fathers (Oliffe, Bottorff, Johnson, Kelly, & LeBeau, 2010) our study also engaged men to detail how they felt (internally and about others) in response to the death of a male peer. In doing so, we confirm photo-elicitation as having great potential to illuminate other men’s health issues including suicide as well as less obvious topics such as fathering and unintentional childhood injury. While claimed here as a strength focussing entirely and exclusively on the experiences of young men might be argued as a limitation. In line with Galdas, Johnson, Percy, and Ratner (2010) we suggest future research might benefit from the inclusion of young women who experience the loss of a peer to empirically unpack gender similarities and differences as a means to further develop youth counselling services.

In conclusion, young men’s risk taking and accidental death is often positioned as an unfortunate fait accompli. By describing the nuanced ways that participants grieved such losses and subsequently constructed masculine identities this innovative study affords opportunities to thoughtfully consider young men’s practices and the potential for catalysing their efforts towards advancing (rather than risking) their health and well-being.

## Figures and Tables

**Photograph 1:**
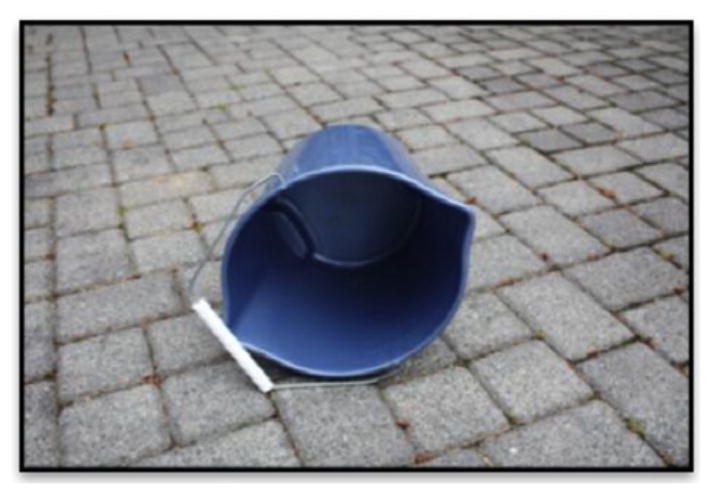
“Empty bucket”.

**Photograph 2:**
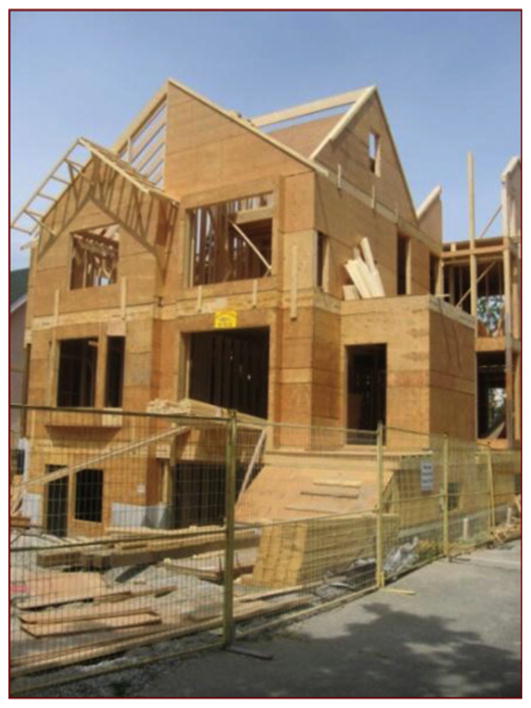
“Incomplete”.

**Photograph 3:**
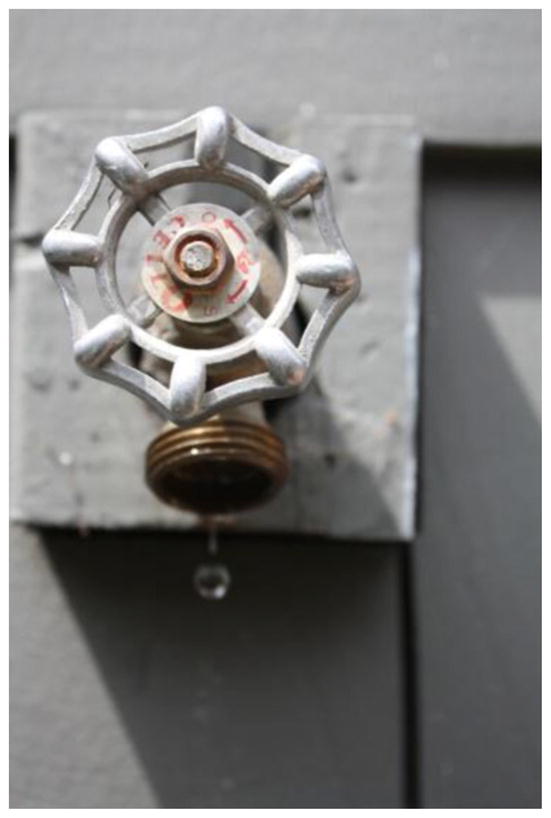
“Turn off the tap”.

**Photograph 4:**
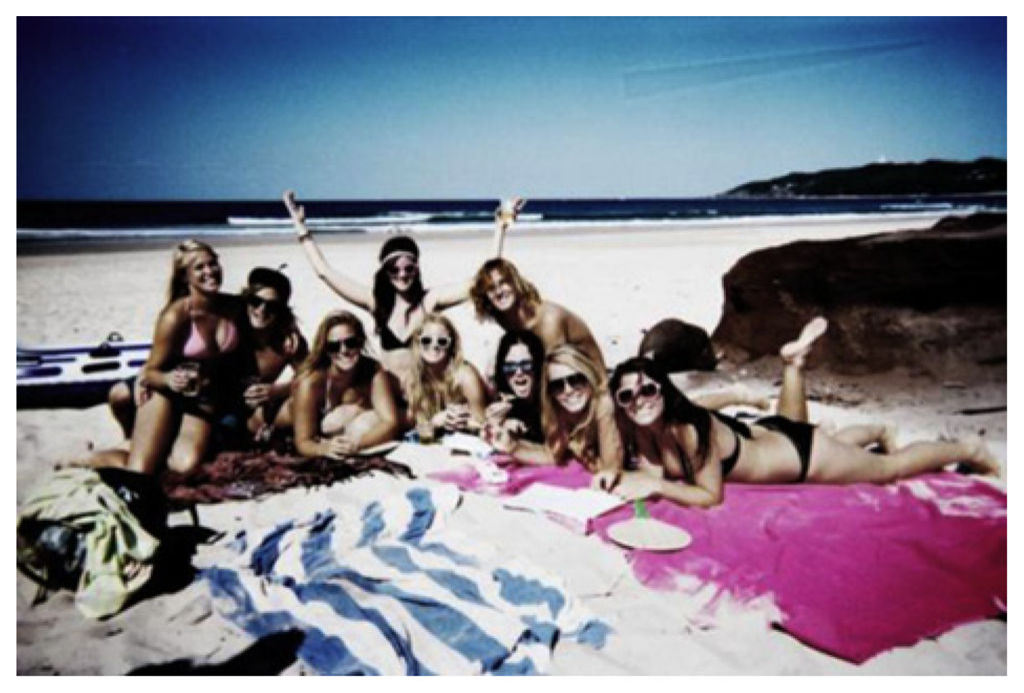
“Beach party”.

**Photograph 5:**
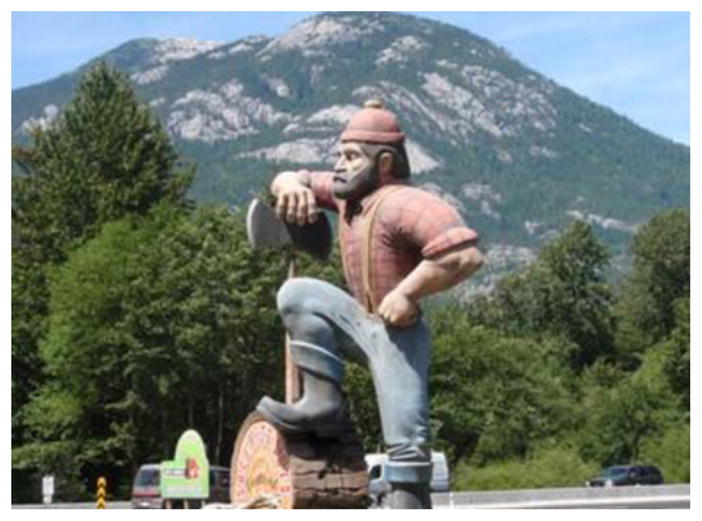
“The lumberjack”.

**Photograph 6:**
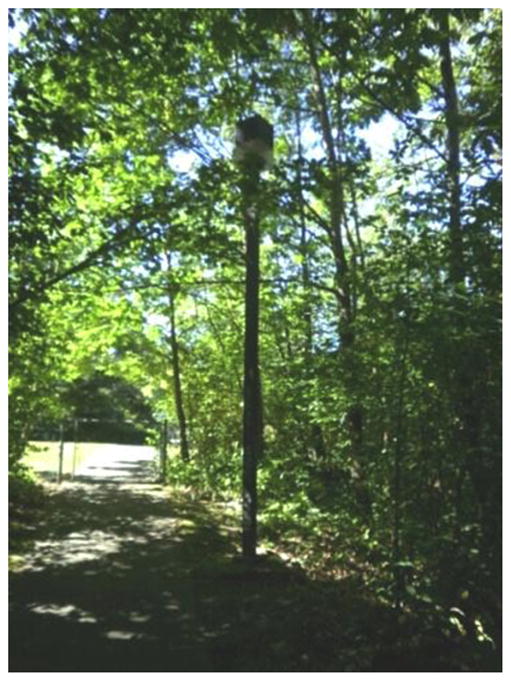
“The lamppost”.

**Table 1:** Participant demographic data.

Age	19–21	21–23	24–25
	7	12	6
Highest education achieved	Some High school	High school Grad	Post-secondary
	7	5	13
Current primary activity	Work	School/work	Unemployed
	11	9	5
Relationship status (all heterosexual)	Single	Dating	Lives with partner
	13	9	3

**Table 2:** Photo assignment prompts.

Where did you and your friend hang out?
What did you and your friend do together?
After your friend died what did you do and where did you go?
What places remind you of your friend?
What do you do to honour your friend?
What picture represents how you felt after your friend died?
What symbolises being a man to you?
What do you think symbolises being a man to your friends?
What does it mean to ‘man up’?
What is one thing you can’t say?
